# SARS-CoV-2 polyprotein expression and the induction of double-membrane vesicles

**DOI:** 10.1128/jvi.01385-25

**Published:** 2025-11-06

**Authors:** Meng Zhao, Yajie Zhang, Yakun Liang, Fuzhi Lei, Yuying Han, Zhenghong Yuan, Zhigang Yi

**Affiliations:** 1 Key Laboratory of Medical Molecular Virology (MOE/NHC/CAMS), Shanghai Institute of Infectious Disease and Biosecurity, Shanghai Frontiers Science Center of Pathogenic Microorganisms and Infection, School of Basic Medical Sciences, Fudan University 58305, Shanghai, China; 2 Shanghai Institute of Precision Medicine, Shanghai Ninth People’s Hospital, Shanghai Jiaotong University School of Medicine 56695, Shanghai, China; 3 Shanghai public health clinical center, Fudan University 34748 , Shanghai, China; University of Kentucky College of Medicine, Lexington, Kentucky, USA

**Keywords:** coronavirus, SARS-CoV-2, double-membrane vesicle, polyprotein cleavage, Nsp6

## Abstract

Coronaviruses induce the formation of double-membrane vesicles (DMVs) to facilitate viral RNA replication and transcription by the replication-transcription complexes (RTCs), comprising non-structural proteins (nsps) 2-16 and nucleocapsid protein. Nsp3 and nsp4 are the minimal components necessary for DMV formation and assemble a molecular pore that spans the DMV double membrane, connecting the DMV interior to the cytosol. However, the recruitment mechanisms of additional RTC components and the roles of other viral proteins in DMV assembly remain poorly understood. To dissect these processes independently of viral replication, we sought to establish a surrogate expression system using severe acute respiratory syndrome coronavirus type 2 (SARS-CoV-2) polypeptides to recapitulate DMV formation and RTC recruitment. We characterized DMVs formed in cells expressing various nsp combinations and assessed the localization of RTC components using proteinase K (PK) protection assays following cell permeabilization. Compared to nsp3-4, expression of nsp3-8 and nsp3-10 resulted in larger and more morphologically heterogeneous DMVs. Portions of nsp5, nsp7, and nsp8 are associated with DMV-enriched membrane fractions, with nsp5 and nsp8 showing partial resistance to proteinase K digestion, suggesting that these proteins are at least partially localized within the DMV interior or protected by the DMV membrane architecture. Notably, mutations in the membrane-associated element (MAE) of nsp6 impaired nsp5-mediated proteolytic processing, abrogated DMV formation, and induced a cross-linked endoplasmic reticulum (ER) phenotype. These results highlight the essential role of nsp6 in the DMV biogenesis and demonstrate the utility of this surrogate system for mechanistic studies of coronavirus-induced membrane remodeling.

**IMPORTANCE:**

Coronaviruses remodel host membranes through the action of non-structural proteins to generate double-membrane vesicles (DMVs), which serve as platforms for viral replication-transcription complexes (RTCs). Deciphering the molecular mechanisms governing DMV assembly and RTC recruitment is critical for understanding coronavirus replication and identifying novel antiviral targets. Here, we developed a surrogate system that recapitulates DMV formation in the absence of viral replication, enabling genetic manipulation and functional dissection of individual proteins. Using this system, we demonstrate that expression of the SARS-CoV-2 nsp3-10 polyprotein is sufficient to drive DMV formation and reveal a pivotal role for the membrane-associated element (MAE) of nsp6 in this process. These findings establish a tractable model for investigating coronavirus-induced membrane remodeling and underscore the essential contributions of nsp6 to DMV biogenesis.

## INTRODUCTION

Coronaviruses are important emerging and re-emerging pathogens. The novel coronavirus pneumonia (coronavirus disease 2019, COVID-19) pandemic caused by severe acute respiratory syndrome coronavirus type 2 (SARS-CoV-2) has posed a severe global social and economic impact (1, 2). Although direct-acting antiviral agents (DAAs), such as viral polymerase inhibitors (3–5) and viral protease inhibitors (6), have shown therapeutic potential in the treatment of COVID-19, there remains a critical unmet need for highly effective DAAs with novel mechanisms of action. The development of such agents would expand and strengthen the current antiviral arsenal, providing improved therapeutic outcomes and broader activity against emerging coronaviruses and their variants.

Coronaviruses are positive-sense, single-stranded RNA viruses. The viral genome encodes an open reading frame ORF1a, producing the polyprotein pp1a (non-structural protein 1-10, nsp1-10). A ribosome-mediated ribosomal frameshift within ORF1a facilitates the translation of ORF1b, leading to the synthesis of the polyprotein pp1ab, which is subsequently processed into non-structural proteins nsp1-16 by two viral cysteine proteases: the papain-like protease (PLpro) and the chymotrypsin-like main protease (3CLpro or Mpro) (7, 8). Coronavirus shares a conserved replication strategy with other single-stranded RNA viruses, assembling a replication complex from viral membrane proteins that serve as platforms for genome replication. The non-structural proteins of SARS-CoV-2 remodel endoplasmic reticulum (ER) to form a double-membrane vesicle (DMV) (9, 10), which is believed to be the place for virus replication and transcription (11, 12).

Coronavirus genome replication and transcription are mediated by the replication-transcription complex (RTC), a multi-protein assembly comprising viral non-structural proteins nsp2 through nsp16 along with the nucleocapsid protein (8, 13–15). This replication-transcription process primarily occurs within DMVs (11–14). SARS-CoV-2 nsp3 and nsp4 constitute the minimal components necessary and sufficient to induce DMV formation (15). These DMVs connect to the host cytoplasm via a pore complex ‌assembled by 12 copies each of nsp3 and nsp4 (16), which is proposed to mediate molecular exchange‌ between the DMV interior and the cytosol (9, 15, 17, 18).

The functional contributions of additional non-structural proteins in DMV biogenesis and the molecular mechanisms governing the recruitment of the RTC components into DMVs remain to be fully elucidated. Moreover, the role of nsp6, a multi-spanning membrane protein, in DMV biogenesis remains incompletely understood and is still a subject of debate. A recent study demonstrated a regulatory role of nsp6 in DMV formation, likely via its involvement in lipid metabolism (19, 20). We recently identified a highly conserved membrane-associated element (MAE) at the C-terminus of nsp6 that is essential for viral replication (21); however, its precise role in DMV biogenesis remains to be elucidated. A recent study demonstrated that, in HCoV-229E-infected cells, the molecular pore forms a large complex with the replicase, including nsp3, nsp4, nsp7, nsp8, nsp9, nsp12, and nsp13, and confirmed that the replicase/transcriptase resides within the DMV interior (18). Recently, by using a co-expression system for SARS-CoV-2 proteins, a report demonstrated that the DMVs assembled by co-expressed nsp3 and nsp4 could recruit the viral RNA polymerase nsp12 via the N-terminal domain of nsp3, suggesting a novel mechanism for the recruitment of the viral replicase/transcriptase complex (22).

To comprehensively elucidate the molecular mechanisms by which viral proteins contribute to DMV biogenesis and RTC components recruitment, a surrogate system for DMV formation distinct from viral replication offers a tractable platform for genetic manipulation of viral proteins and subsequent phenotypic analysis of DMV development. In this study, we established a system capable of expressing polyproteins encompassing nsp3-10 and observed the resultant DMV formation. Furthermore, leveraging this polyprotein expression system, we delineated the roles of the MAE of nsp6 in DMV assembly. This system would provide a robust platform for dissecting the processes underlying DMV assembly and determining the structural characteristics of DMVs harboring components of the viral RTCs.

## RESULTS

### Establishment of a SARS-CoV-2 polyprotein expression system

HEK293T cells were selected for the viral polyprotein expression system due to their high plasmid transfection efficiency, enabling robust delivery of expression constructs. We first evaluated the capability of HEK293T cells for supporting SARS-CoV-2 replication. HEK293T cells were co-transfected with *in vitro*-transcribed SARS-CoV-2 replicon RNAs and mRNA encoding the nucleocapsid (N) protein following an established protocol (23). Replication of the replicon RNA was confirmed by a marked increase in luciferase activity, in contrast to the non-replicative control bearing an inactive mutation (SAA) in the RNA-dependent RNA polymerase (Fig. S1A), comparable with the replication kinetics observed in Huh7 cells (Fig. S1B). These results demonstrate that HEK293T cells are permissive for SARS-CoV-2 RNA replication, supporting the physiological relevance of this system for viral polyprotein expression.

**Fig 1:**
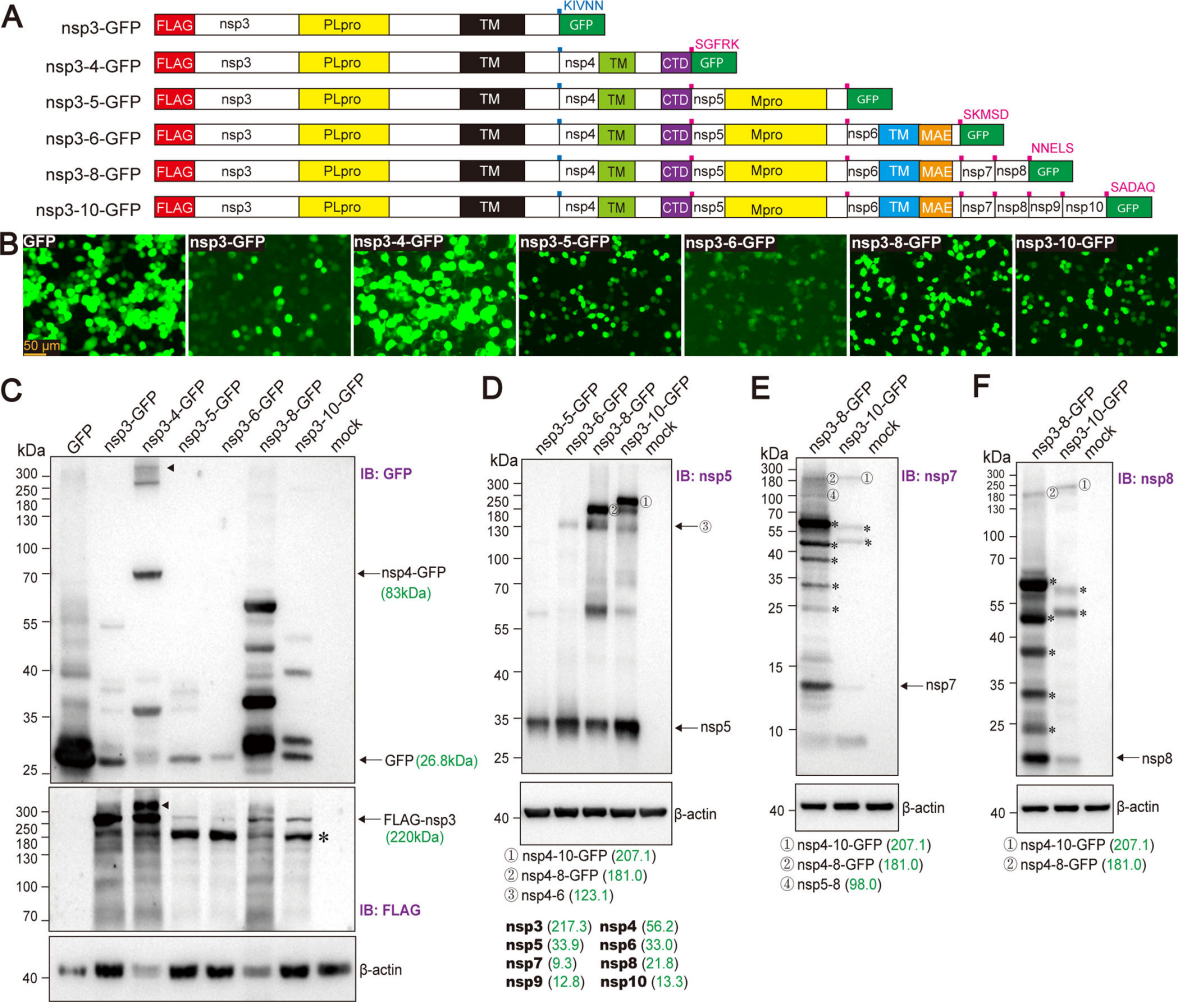
Expression profiles of SARS-CoV-2 polyproteins. (**A**) Schematic of SARS-CoV-2 expression constructs driven by a CMV promoter. The GFP sequence is flanked by duplicated cleavage sites; TM indicates transmembrane regions. (**B**) Fluorescence microscopy of HEK293T cells transfected with the indicated plasmids, imaged at 48 h post-transfection. (**C–F**) Western blot analysis of HEK293T cells transfected as indicated. At 48 h post-transfection, samples were collected as described in Materials and Methods without prior boiling and analyzed by immunoblotting (IB) with the specified antibodies. A representative image from multiple independent experiments is shown. Putative polyprotein cleavage intermediates are annotated with predicted molecular weights. Molecular weight markers (kDa) are shown at the left. Theoretical molecular weights of proteins are shown in parentheses. Asterisks in panels **C**, **E**, and **F** indicate unspecified protein bands, and diamonds in panel **C** denote the putative nsp3-4-GFP precursor.

To express SARS-CoV-2 polyproteins, viral open reading frames (ORFs) encoding various combinations of non-structural proteins, including nsp3, nsp3-4, nsp3-5, nsp3-6, nsp3-8, and nsp3-nsp10, were cloned downstream of a cytomegalovirus (CMV) promoter in a low-copy plasmid, pLCCMV. This vector was selected to facilitate the stable cloning of large viral DNA fragments, as described in Materials and Methods. For detection, a 3×FLAG tag was inserted at the N-terminus of nsp3, a modification that we have previously shown to be compatible with viral replication (24). To monitor expression and processing, a green fluorescent protein (GFP) was fused to the C-terminus of each polyprotein via a linker peptide that restores the native viral cleavage site (Fig. 1A). Upon expression in cells, the polyproteins are processed by the viral protease: nsp3 (PLpro) at the nsp3/4 junction, and nsp5 (Mpro) at the nsp4/5, nsp5/6, nsp6/7, nsp7/8, nsp8/9, nsp9/10, and the nsp/GFP junctions (Fig. 1A). Cleavage of GFP serves as an indicator of polyprotein expression and processing efficiency. Following transient transfection into HEK293T cells, all constructs were expressed and typically yielded over 20% GFP-positive cell population with varying levels of GFP fluorescence (Fig. S2; Fig. 1B). The observed variability in GFP signal likely reflects differences in the expression levels and/or stability of the corresponding polyproteins. Western blot analysis of the cell lysates was performed to confirm the expression of FLAG-tagged nsp3 and the proteolytic cleavage of GFP from the polyprotein constructs. In particular, processing of the nsp3-4-GFP construct resulted in the expected nsp4-GFP product. Cleaved GFP was also detected in cells transfected with nsp3-GFP, nsp3-5-GFP, nsp3-6-GFP, nsp3-8-GFP, and nsp3-nsp10-GFP constructs. Notably, the expression level of FLAG-tagged nsp3, as detected by anti-FLAG immunoblotting, was substantially reduced in cells transfected with the nsp3-5-GFP, nsp3-6-GFP, nsp3-8-GFP, and nsp3-nsp10-GFP constructs, compared to those transfected with nsp3-GFP or nsp3-4-GFP, indicating lower polyprotein expression levels and/or protein stability (Fig. 1B). Additionally, multiple bands were observed in both anti-GFP and anti-FLAG blots, which likely represent processing intermediates or degradation products (Fig. 1C).

To further investigate polyprotein processing in cells transfected with various constructs, western blot analysis was performed using antibodies against nsp5, nsp7, and nsp8. Anti-nsp6 antibody was excluded due to poor specificity. The specificity of the selected antibodies was validated by expressing individual affinity-tagged viral proteins, followed by parallel immunoblotting with both the commercially available antibodies and anti-tag antibodies (Fig. S3).

The expression and processing of nsp5 were analyzed in HEK293T cells transfected with the nsp3-5-GFP, nsp3-6-GFP, nsp3-8-GFP, and nsp3-10-GFP constructs. Western blot analysis revealed efficient processing of nsp5 in all conditions. The identities of the protein bands were inferred based on their predicted molecular weights. Notably, two prominent high-molecular-weight bands were detected in cells transfected with nsp3-8-GFP and nsp3-10-GFP constructs (Fig. 1D). These bands corresponded to the predicted sizes of nsp4-10-GFP (~207.1 kDa) and nsp4-8-GFP (~181.0 kDa), respectively. A third band, observed across cells transfected with nsp3-6-GFP, nsp3-8-GFP, and nsp3-10-GFP, matched the predicted molecular weight of nsp4-6 (~123.1 kDa) (Fig. 1D). Immunoblotting with anti-nsp7 and anti-nsp8 antibodies further confirmed the identities of the nsp4-8-GFP and nsp4-10-GFP intermediates (Fig. 1E and F). The accumulation of these precursors suggests inefficient cleavage of the nsp4-8 and nsp4-10, consistent with prior observations in murine hepatitis virus-infected cells, where a similar ~150 kDa intermediate corresponding to nsp4-10 was reported (25–28). In nsp3-8-GFP- and nsp3-10-GFP-transfected cells, several unspecified protein bands with molecular weights of approximately 25–75 kDa were observed. Given that cleavage at the nsp7/8 junctions represents a rate-limiting step (29), the observed bands likely correspond to putative processing intermediates, including nsp7-8-GFP (~57.9 kDa) and nsp8-GFP (~48.6 kDa) in nsp3-8-GFP-expressing cells and nsp7-10 (~57.2 kDa) and nsp8-10 (~47.9 kDa) in nsp3-10-GFP-expressing cells (Fig. 1E and F).

To further investigate these processing intermediates, we introduced catalytically inactive mutations in nsp3 (PLpro.C856A) and nsp5 (Mpro.C145A) into the 3-8-GFP construct (Fig. S4A). Western blot analysis of these mutants showed that processed nsp5 and nsp8 were absent in cells expressing 3-8-GFP.C145A, but readily detected in cells expressing either wild-type 3-8-GFP (WT) or 3-8-GFP.C856A (Fig. S4B). These results indicate that Mpro-mediated cleavage proceeds independently of PLpro activity at the nsp3/4 junction. Notably, a high-molecular-weight band corresponding to nsp4-8-GFP intermediate was present in the anti-nsp5 blot for both wild-type and C145A mutant-expressing cells, with enhanced intensity in the latter. This nsp4-8-GFP band was faint in the anti-nsp8 blot, likely due to the lower sensitivity of the anti-nsp8 antibody (Fig. S4B). Notably, the unspecified bands previously suspected to represent nsp7-8 intermediates in nsp3-8-GFP-expressing cells, as detected with anti-nsp8 antibody, were also readily observed in the nsp3-8-GFP.C145A-expressing cells, in which Mpro-mediated cleavage is expected to be absent (Fig. 1F; Fig. S4B). This observation suggests that these bands more likely correspond to degradation products rather than authentic nsp7-8 intermediates. Nevertheless, the data support the accumulation of polyprotein processing intermediates, including at least the nsp4-8 species.

In summary, we established that SARS-CoV-2 polyproteins are expressed and proteolytically processed in HEK293T cells, revealing distinct processing patterns and intermediate species consistent with expected cleavage kinetics.

### Formation of DMVs induced by SARS-CoV-2 polyprotein expression

**Fig 2:**
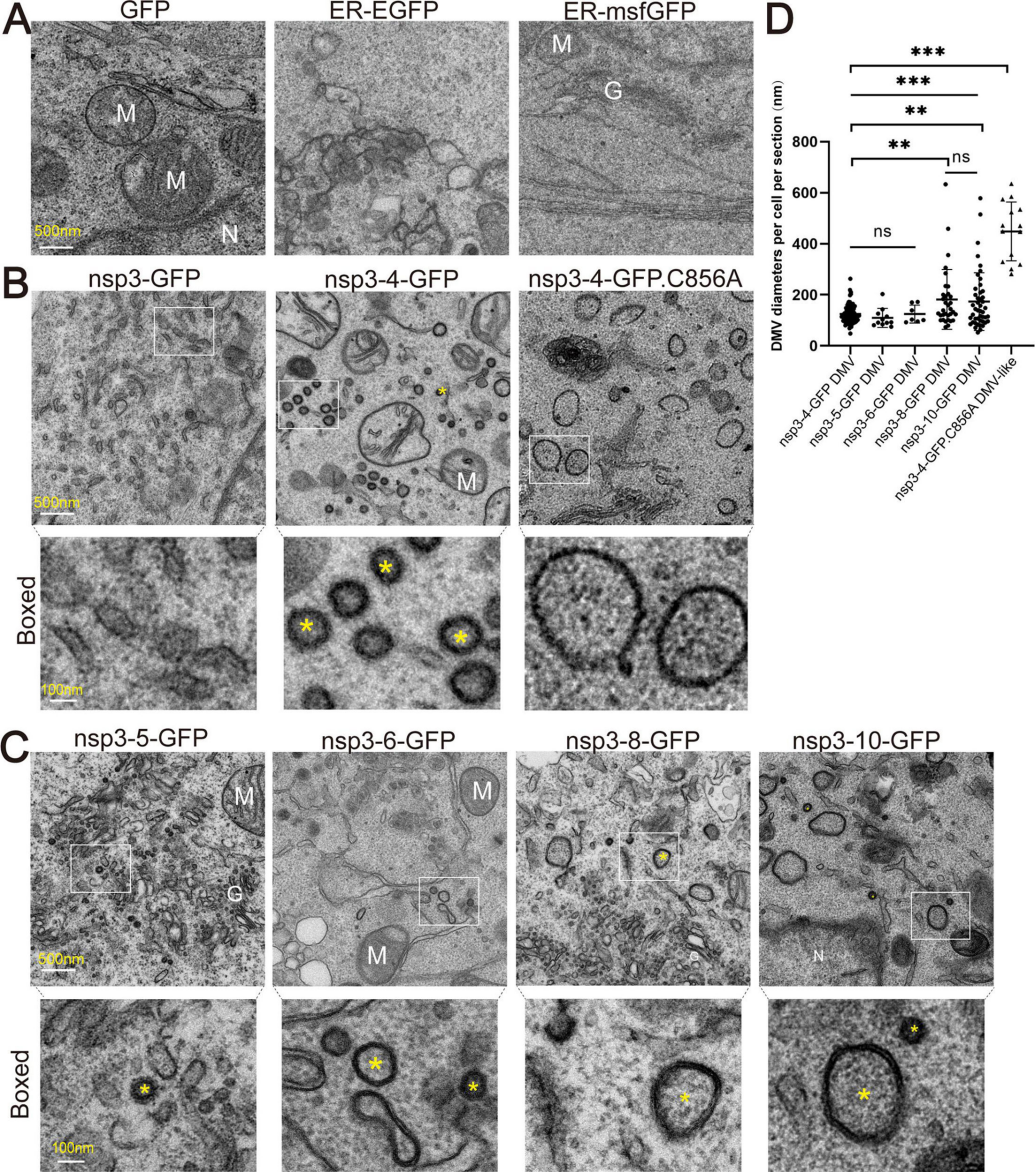
DMV formation induced by SARS-CoV-2 polyprotein expression. (**A–C**) HEK293T cells transfected with the indicated plasmids were analyzed by electron microscopy at 48 h post-transfection. Scale bars: 500 nm; magnified boxed regions, 100 nm. Representative DMVs are indicated by yellow asterisks. N, nucleus; G, Golgi; M, mitochondria. Constructs include an N-terminal 3×Flag tag and a C-terminal GFP tag. (**D**) Quantification of DMV diameters per cell (n) per section (N) (mean ± SEM) from indicated constructs, based on 2–6 independent experiments: nsp3-4-GFP (*N* = 3, *n* = 82), nsp3-5-GFP (*N* = 8, *n* = 11), nsp3-6-GFP (*N* = 12, *n* = 7), nsp3-8-GFP (*N* = 4, *n* = 33), nsp3-10-GFP (*N* = 4, *n* = 46), and nsp3-4-GFP.C856A (*N* = 4, *n* = 14). Statistical significance was evaluated by one-way ANOVA with Dunnett’s multiple comparisons test (control: nsp3-4-GFP) and unpaired two-tailed *t*-test where indicated. ***, *P* < 0.001; **, *P* < 0.01; ns, not significant.

To investigate the formation of DMVs, HEK293T cells were transfected with SARS-CoV-2 polyprotein constructs. Constructs encoding GFP, ER-targeted GFP (ER-GFP), and ER-targeted monomeric superfolder GFP (ER-msfGFP) (30) were used as controls, representing a cytosolic non-specific protein, an ER-associated oligomeric protein, and an ER-associated monomeric non-specific protein, respectively. Ultrathin sections of the fixed cells were analyzed using transmission electron microscopy (TEM). No DMVs were observed in cells expressing GFP, ER-GFP, or msfGFP. However, ER sheet alterations were evident in ER-GFP-expressing cells, but not in ER-msfGFP-expressing cells (Fig. 2A), likely reflecting the oligomerization of GFP on the ER membrane, in contrast to the monomeric msfGFP.

Formation of DMVs was not observed in cells expressing nsp3-GFP, whereas they were readily detected in cells expressing nsp3-4-GFP (Fig. 2B), consistent with prior studies demonstrating that nsp3 alone is insufficient to drive DMV biogenesis (15, 31). These results confirm that the cleaved forms of nsp3 and nsp4-GFP are functionally competent for DMV assembly, supporting recent findings that nsp4 C-terminal fusions can support DMV formation (22).

Cleavage at the nsp3/4 junction is known to be essential for DMV formation. In contrast to previous cryo-electron tomography studies showing that mutations at the nsp3/4 cleavage site result in multilayered paired-membrane structures instead of DMVs (15, 16), we observed vesicle-like and DMV-like structures with significantly enlarged diameters in cells expressing the catalytically inactive PLpro mutant construct (nsp3-4-GFP.C856A) (Fig. 2B and D). However, due to the limited resolution of TEM, the precise morphology of these structures could not be definitively determined.

While nsp3 and nsp4 are the minimal viral components required for DMV assembly (15), the potential contributions of additional non-structural proteins remain to be elucidated. We therefore examined DMV formation in cells expressing extended polyproteins: nsp3-5, nsp3-6, nsp3-8, and nsp3-10. All constructs supported the formation of DMVs (Fig. 2C). The average diameters of DMVs formed in cells expressing nsp3-4-GFP, nsp3-5-GFP, and nsp3-6-GFP were comparable, measuring 125.0 ± 3.9 nm, 109.5 ± 11.2 nm, and 124.8 ± 13.3 nm, respectively. These values are consistent with those previously reported for DMVs induced by nsp3-4 expression (14–16). In contrast, expression of nsp3-8-GFP and nsp3-10-GFP produced DMVs with significantly larger diameters (181.9 ± 30.3 nm and 167.8 ± 16.7 nm, respectively) and greater heterogeneity, as evidenced by a wider distribution of diameters (Fig. 2C and D). These values approach the size of DMVs in infected cells (~250 nm) (32).

Together, these findings suggest that additional non-structural proteins, particularly nsp7-10, may influence DMV size and morphology, potentially by modulating membrane architecture.

### Characterization of DMVs in SARS-CoV-2 polyprotein-expressing cells

**Fig 3:**
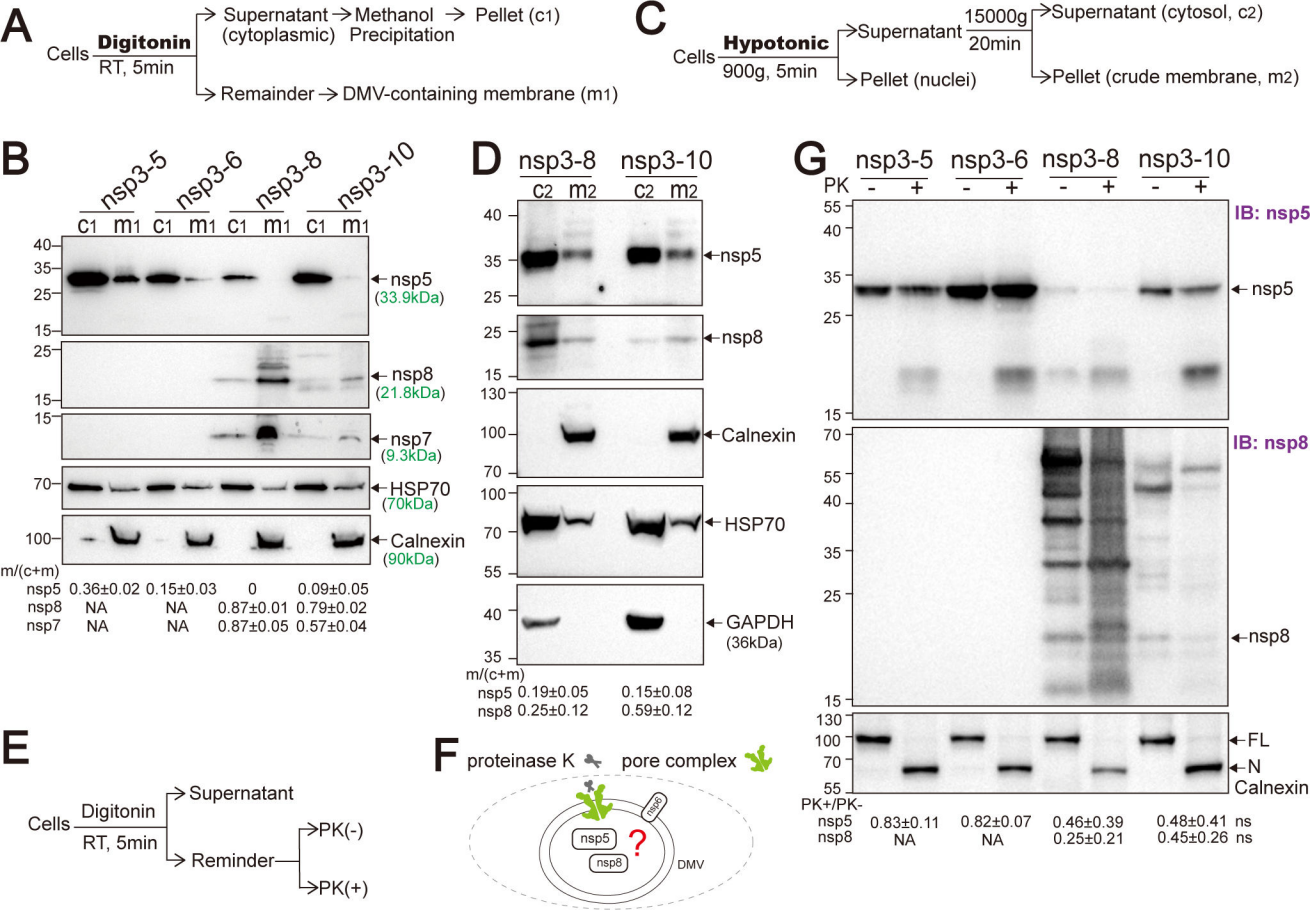
Characterization of DMVs in cells expressing SARS-CoV-2 polyproteins. (**A**) Schematic of fractionation protocol 1: digitonin permeabilization to isolate cytosolic (c1) and membrane (m1) fractions. Cytosolic proteins were methanol-precipitated and resuspended in SDS loading buffer; the membrane fraction was scraped directly into SDS loading buffer. (**B**) Western blot analysis of c1 and m1 fractions from HEK293T cells transfected with the indicated plasmids at 48 h post-transfection, probed for nsp5, nsp7, and nsp8. Protein distribution ratios were quantified (mean ± SEM, *n* = 3). (**C**) Schematic of fractionation protocol 2: hypotonic buffer permeabilization, low-speed centrifugation to remove debris, followed by high-speed centrifugation to separate cytosolic (c2) and membrane (m2) fractions. (**D**) Western blot analysis of c2 and m2 fractions from transfected HEK293T cells at 48 h, probed for nsp5, nsp8, Calnexin, Hsp70, and GAPDH. Protein distribution ratios quantified (mean ± SEM, *n* = 3). (**E**) Schematic of proteinase K digestion following digitonin permeabilization: cytosolic fraction removed, remaining cellular material digested with or without proteinase K, proteins precipitated by TCA, resuspended in SDS loading buffer for analysis. (**F**) Model illustrating the localization of non-structural proteins within DMVs; digitonin permeabilization leaves DMV membranes intact for protease protection assays. (**G**) Proteinase K protection assay on HEK293T cells expressing nsp3-5-GFP, nsp3-6-GFP, nsp3-8-GFP, or nsp3-10-GFP. Proteinase K digestion was performed at 48 h post-transfection; Western blotting for nsp5 and nsp8 was quantified by ImageJ. Protection rates were calculated by normalizing proteinase K (PK)-treated to untreated band intensities (mean ± SEM, *n* = 3–7). FL, full-length Calnexin; N, N-terminal Calnexin. Statistical significance was evaluated by an unpaired two-tailed *t*-test; ns, not significant.

To further characterize DMVs formed upon SARS-CoV-2 polyprotein expression, we examined the subcellular distribution of non-structural proteins nsp5, nsp7, and nsp8, which lack intrinsic membrane-targeting motifs. Cellular fractionation was performed following digitonin treatment, which selectively permeabilizes the plasma membrane while preserving intracellular membrane structures, including DMVs. The resulting cytosolic supernatant (c1) and the remaining cellular material, which includes DMV-associated membrane (m1) (18), were collected for analysis (Fig. 3A). Western blotting was used to evaluate protein distribution across fractions. Calnexin, an ER marker, was enriched in the membrane fraction. HSP70, a cytoplasmic chaperone protein, was predominantly found in the cytosolic fraction, although a minor portion was present in the membrane fraction (Fig. 3B), likely due to interactions with membrane-associated complexes.

We next assessed the distribution of non-structural proteins across these fractions. In cells expressing the nsp3-5-GFP polypeptide, approximately 36% of nsp5 was detected in the membrane fraction. This proportion decreased to 15% and 9% in cells expressing nsp3-6-GFP and nsp3-10-GFP, respectively, and nsp5 was undetectable in the membrane fraction of nsp3-8-GFP-expressing cells under these conditions. These findings suggest that the membrane association of nsp5 may be influenced by the presence or absence of other viral proteins, such as nsp9 and nsp10. In contrast, nsp7 and nsp8 exhibited a stronger membrane association. In nsp3-8-GFP-expressing cells, approximately 87% of both nsp7 and nsp8 were detected in the membrane fraction. Similarly, in cells expressing nsp3-10-GFP, 57% of nsp7 and 79% of nsp8 localized to the membrane. These findings demonstrate that, although nsp5, nsp7, and nsp8 lack membrane-targeting sequences, they associate with membranes, likely through interactions with membrane-integrated viral components (Fig. 3B).

To validate these findings and further resolve nsp5 localization, an alternative cellular fractionation method using a larger number of cells was employed, as described in Materials and Methods. Cells were lysed in a hypotonic buffer. The post-nuclear supernatants were subjected to differential centrifugation to separate the cytosolic fraction (c2) and a crude membrane fraction (m2), which is enriched in DMVs (33). Under these conditions, 19% and 15% of nsp5 and 25% and 59% of nsp8 were found in the membrane fraction in cells expressing nsp3-8-GFP and nsp3-10-GFP, respectively (Fig. 3D). Similar to the digitonin-based fractionation, HSP70 was partially detected in the membrane fraction. Glyceraldehyde 3-phosphate dehydrogenase (GAPDH), a cytosolic marker, was exclusively located in the membrane fraction (Fig. 3D).

To elucidate the topological localization of nsp5, nsp7, and nsp8 within DMV, we employed proteinase K (PK), a broad-spectrum serine protease, to digest membrane fractions following digitonin-mediated permeabilization. The luminal compartment of DMVs is protected from PK-mediated proteolysis (33, 34). Exogenous proteins fused to the C-terminus of nsp4 exhibit partial resistance to PK digestion (18), likely due to the size restriction imposed by the nsp3-4-formed central pore (~17 Å) (16) or the DMV membrane architecture, which excludes larger cytosolic proteins, such as PK (~67 Å). This selective protease protection provides a basis for inferring the subcellular localization of proteins relative to the DMV structure based on their susceptibility to PK treatment. Proteins residing within the DMV interior or associated with the DMV membrane architecture are shielded from digestion and thus retain their integrity upon PK treatment (Fig. 3E and F). The efficiency of PK digestion was validated using calnexin, an ER marker containing a large N-terminal luminal domain, as a control. Western blot analysis confirmed its protection pattern, consistent with luminal localization (Fig. 3G). In cells expressing the polyproteins, nsp5 was protected from PK digest by 83% ± 11%, 82% ± 7%, 46% ± 39% and 48% ± 41% in nsp3-5-GFP-, nsp3-6-GFP-, nsp3-8-GFP-, and nsp3-10-GFP-expressing cells, respectively, suggesting that the majority of membrane-associated nsp5 is protected by DMV structure. In contrast, in cells expressing nsp3-8-GFP and nsp3-10-GFP, the protection levels of nsp5 were reduced to 9% and 21%, respectively. Similarly, nsp8 exhibited protection rates of 25% ± 21% and 45% ± 26% in nsp3-8-GFP- and nsp3-10-GFP-expressing cells, respectively (Fig. 3G). These findings suggest a substantial portion of nsp5 and nsp8 is localized within the DMV interior or shielded by the DMV membrane architecture.

In summary, we established a SARS-CoV-2 polyprotein expression system capable of inducing DMV assembly and demonstrated that nsp5 and nsp8 can be recruited to the DMV. These findings are consistent with previous observations showing that, although nsp5 and nsp8 exhibit diffuse cytoplasmic localization when expressed individually, they adopt a punctate distribution that colocalizes with double-stranded RNA (dsRNA) in SARS-CoV-2-infected cells, indicating their recruitment to DMV structures (35).

### Mutation of nsp6 membrane-associated element impairs DMV formation

**Fig 4:**
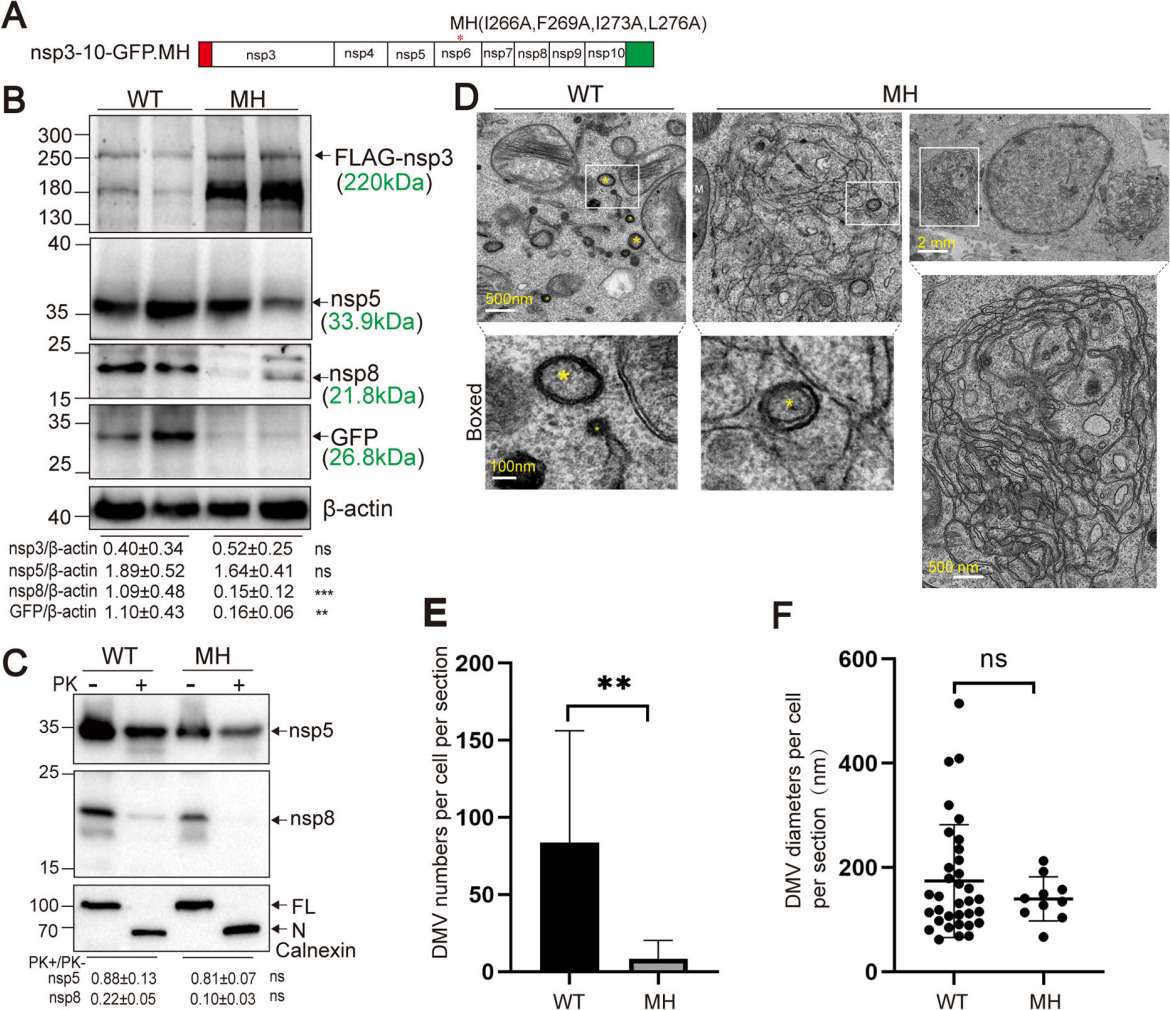
The nsp6 MH mutant attenuates DMV formation. (**A**) Schematic of constructs. The MH mutant carries I266A, F269A, I273A, and L276A mutations. Red bar: 3×Flag tag; green box: GFP. (**B**) Western blot analysis of HEK293T cells transfected with nsp3-10-GFP (WT) or nsp3-10-GFP.MH (MH) at 48 h post-transfection, probed with indicated antibodies. Band intensities for nsp3, GFP, nsp5, and nsp8 were quantified and normalized to Actin (mean ± SEM, *n* = 4–7; unpaired two-tailed *t*-test); ns, not significant; **P < 0.01; ***P < 0.001. (**C**) Proteinase K protection assay following transfection with WT or MH constructs. Western blotting for nsp5, nsp8, and Calnexin; band intensities quantified and normalized to untreated controls (mean ± SD, *n* = 3; unpaired two-tailed *t*-test); ns, not significant. FL, full-length Calnexin; N, N-terminal fragment. (**D**) Electron microscopy of transfected cells at 48 h. DMVs are marked with yellow asterisks; M, mitochondria. Scale bars: 500 nm; magnified boxed regions, 100 nm. *N* = 3 for WT; *N* = 4 for MH. (**E**) Quantification of DMV numbers per cell (n) per section (N) from two independent experiments (mean ± SEM; *N* = 3, *n* = 12 for WT; *N* = 4, *n* = 6 for MH). **P < 0.01. (**F**) Quantification of DMV diameters per cell (n) per section (N) from two independent experiments (mean ± SEM; *N* = 3, *n* = 34 for WT; *N* = 4, *n* = 10 for MH; Mann–Whitney test); ns, not significant.

The involvement of the nsp6 protein in DMV biogenesis has been a subject of ongoing debate. Recent studies suggest that nsp6 plays a regulatory role in this process, potentially through modulation of lipid metabolism (19, 20). In a previous study, we identified a highly conserved MAE at the C-terminal region of nsp6 that is essential for viral replication (21). However, the specific role of the MAE in DMV formation remains unclear. We used the polyprotein expression system described above, which includes nsp6, to examine the impact of the MAE on DMV assembly. To this end, a combination of inactivating mutations of the nsp6 MAE (MH: I266A, F269A, I273A, L276A) (21) was introduced into the nsp3-10-GFP construct, yielding the nsp3-10-GFP.MH plasmid (Fig. 4A). Expression of viral proteins was first evaluated by western blot. The MH mutation led to a significant reduction in the expression levels of nsp8 and GFP, while the level of nsp3 and nsp5 remained comparable to that of wild-type control (Fig. 4B). To assess whether the reduced expression was due to impaired nsp6 protein stability, we analyzed expression from an HA-tagged nsp6 construct containing the same MH mutations (21). Western blot analysis showed that MH mutations did not significantly alter nsp6 protein levels compared to wild-type HA-nsp6 (Fig. S5), indicating that the mutations likely affect proteolytic processing rather than protein stability. These data suggest that the MH mutations selectively attenuate cleavage at the junctions located downstream of nsp6.

We next investigated the effect of the nsp6 MH mutation on the protease accessibility of nsp5 and nsp8. Cells expressing either wild-type nsp3-10-GFP or nsp3-10-GFP.MH were subjected to digitonin permeabilization, followed by PK digestion. Western blot analysis revealed that 81% of nsp5 remained protected in cells expressing the MH mutant, compared to 88% in wild-type-expressing cells. Similarly, 10% of nsp8 was protected in the MH condition, compared with 22% in the wild type. Quantitative analysis revealed no statistically significant differences in PK sensitivity between the two groups (Fig. 4C), suggesting that the nsp6 MH mutation does not substantially alter the membrane protection of nsp5 and nsp8.

Finally, we evaluated DMV formation in cells expressing either nsp3-10-GFP.MH or nsp3-10-GFP (WT) by TEM. Although DMVs were observed in both conditions, their abundance was significantly reduced in MH-expressing cells, with an average of 8 per cell compared to 83 per cell in wild-type-expressing cells (Fig. 4D and E). The average DMV diameter in MH-expressing cells (139.9 ± 13.3 nm) was comparable to that in wild-type-expressing cells (170.0 ± 18.6 nm) (Fig. 4D and F). Notably, MH-expressing cells exhibited cross-linked ER structures, which were absent in wild-type-expressing cells (Fig. 4D). Given the comparable PK protection of nsp5 and nsp8 in both groups (Fig. 4C), the protection observed in MH-expressing cells likely reflects sequestration within the cross-linked ER rather than genuine DMV formation.

Taken together, these findings indicate that the nsp6 MAE is critical for efficient DMV formation. The MH mutations markedly impair Mpro-mediated processing, reduce DMV abundance, and induce aberrant membrane rearrangements, highlighting a role for the C-terminal region of nsp6 in coordinating polyprotein cleavage and virus-induced membrane remodeling (see Discussion).

### Aberrant polyprotein processing and DMV formation

**Fig 5:**
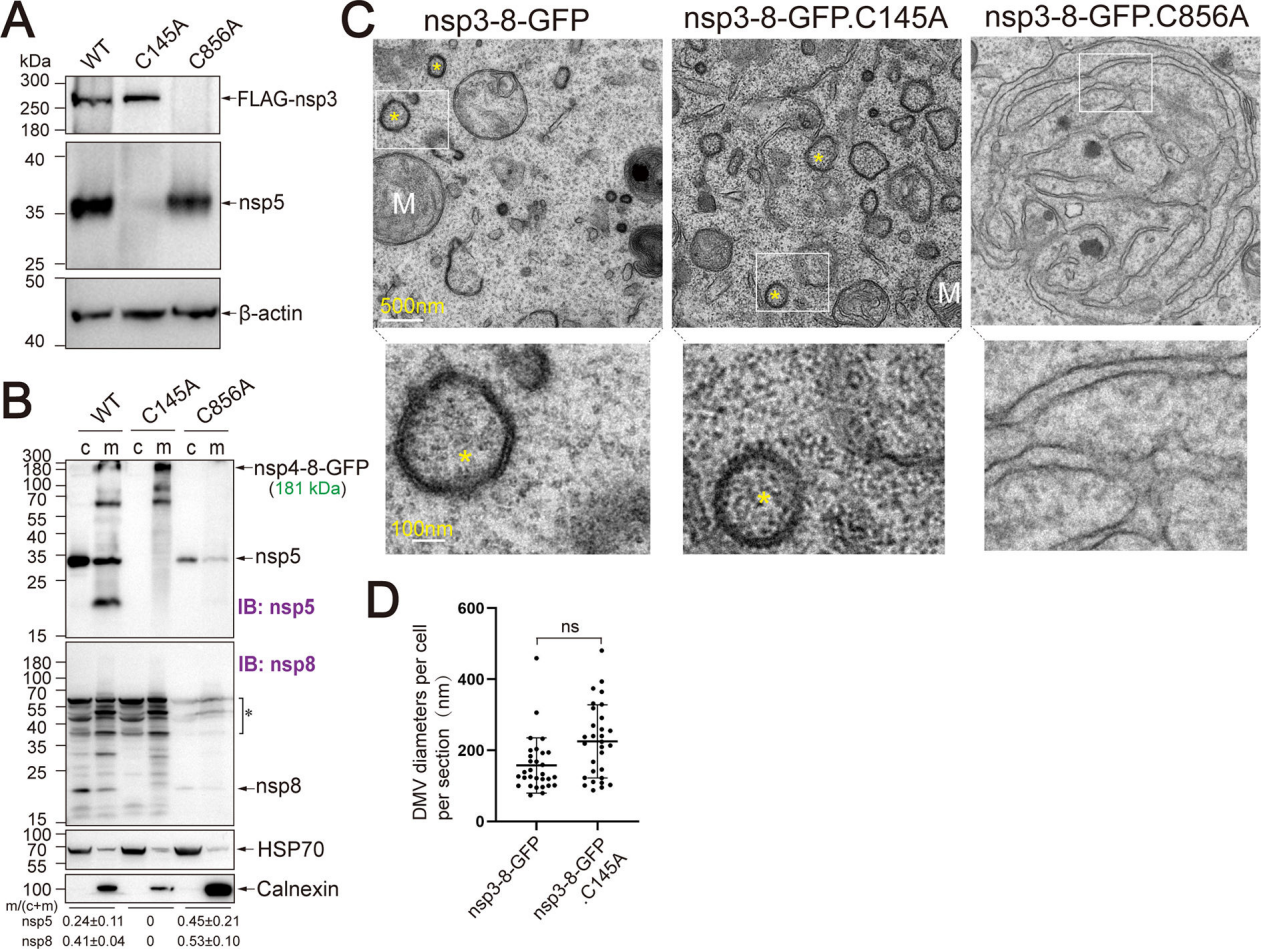
DMV formation by nsp3 PLpro and nsp5 Mpro mutant polyproteins. (**A**) Western blot of HEK293T cells transfected with nsp3-8-GFP (WT), nsp3-8-GFP.C856A (nsp3 PLpro), and nsp3-8-GFP.C145A (nsp5 Mpro) at 48 h post-transfection, probed with indicated antibodies. Representative blot shown; molecular weight markers (kDa) on left. (**B**) Western blot analysis of cytoplasmic (c) and membrane (m) fractions isolated as in Fig. 3A from transfected cells. Samples were collected as described in Materials and Methods without prior boiling and analyzed by immunoblotting (IB). Specific protein bands are indicated by arrows. Unspecified bands are marked with asterisks. Band intensities were quantified, and membrane-to-cytosol distribution ratios calculated (mean ± SEM, *n* = 3). (**C**) Electron microscopy of transfected cells at 48 h. Scale bars: 500 nm; magnified boxed regions, 100 nm. Representative DMVs indicated by yellow asterisks; M, mitochondria (*N* = 4 for C856A; *N* = 2 for WT and C145A). (**D**) Quantification of DMV diameters per cell (n) per section (N) from two independent experiments (mean ± SEM; *N* = 4 for C856A; *N* = 2, *n* = 30 for C145A). Statistical significance assessed by unpaired two-tailed *t*-test; ns, not significant.

Given that MH mutations impair Mpro-mediated processing and induce aberrant membrane rearrangements, thereby reducing DMV formation, it remains unclear whether Mpro-mediated processing is strictly required for DMV assembly. Previous studies have shown that cleavage between nsp3 and nsp4 is essential for DMV formation in the nsp3-4 expression system (15, 16). However, in the context of extended polyproteins, the individual contributions of PLpro- and Mpro-mediated cleavage events to DMV biogenesis have yet to be elucidated. To investigate the role of proteolytic processing in DMV formation, we employed catalytically inactive mutants: nsp3-8-GFP.C856A, harboring a PLpro-inactivating mutation in nsp3 and nsp3-8-GFP.C145A, harboring an Mpro-inactivating mutation in nsp5 (Fig. S4A). The nsp5.C145A mutation abolished Mpro activity, as indicated by the absence of cleaved nsp5 and nsp8 bands (Fig. 5A; Fig. S4B). Nevertheless, nsp3/4 cleavage remained unaffected, with cleaved nsp3 bands detected at levels comparable to the wild-type control (Fig. 5A), alongside an accumulation of uncleaved nsp4-8-GFP (Fig. S4B), suggesting that nsp3/4 cleavage occurs independently of nsp5-mediated processing. Conversely, the C856A mutation did not impair Mpro-mediated cleavage, as evidenced by the presence of cleaved nsp5 in cells expressing nsp3-4-GFP.C856A (Fig. 5A). Notably, uncleaved nsp3-4 species were not detected in cells expressing nsp3-8-GFP.C856A, likely due to the intrinsic instability of the uncleaved polyprotein within the extended nsp3-8 context. However, uncleaved nsp3-4-GFP was readily observed in cells expressing the shorter nsp3-4-GFP.C856A (Fig. S6), suggesting that polyprotein stability may vary depending on construct length and context.

Then we assessed the membrane association of polyproteins carrying the C856A and C145A mutations following digitonin-mediated permeabilization, as described previously (Fig. 3A). In the cells expressing nsp3-8-GFP.C145A, the uncleaved nsp4-8-GFP was exclusively detected in the membrane fraction, similar to the ER marker calnexin. This is likely attributable to the presence of integral membrane proteins nsp4 and nsp6 within the unprocessed polyprotein. In cells expressing nsp3-8-GFP.C856A, a portion of the processed nsp5 and nsp8 was detected in the membrane fraction, comparable to the distribution observed in cells expressing wild-type nsp3-8-GFP (Fig. 5B). These results suggest that the membrane association of nsp5 and nsp8 is independent of nsp3/4 cleavage.

Finally, we examined DMV formation in cells expressing the mutant polyproteins. In cells expressing nsp3-8-GFP.C145A, DMV structures were observed with diameters comparable to those formed by wild-type nsp3-8-GFP, although increased morphological heterogeneity was noted (Fig. 5C and D). In contrast, no DMVs were detected in cells expressing nsp3-8-GFP.C856A; instead, extended membrane sheets were observed (Fig. 5C). These findings indicate that nsp3/4 cleavage is essential for DMV formation in the context of extended polyproteins, whereas Mpro-mediated processing is dispensable (see Discussion).

## DISCUSSION

Coronaviruses assemble DMVs that serve as compartments for viral genome replication and transcription, mediated by the RTCs (11–14). A molecular pore, assembled by nsp3 and nsp4, spans the DMV membranes and connects the DMV interior and the cytosol (9, 16). The RTC comprises viral non-structural proteins nsp2-16 together with the nucleocapsid protein (8, 36–38). High-resolution structures have been determined for a core viral replicase complex composed of nsp7, nsp8, nsp12, and RNA, as well as for a larger complex containing nsp7, nsp8, nsp12, RNA, nsp9, and nsp13 (37, 39, 40). Recent work in HCoV-229E-infected cells revealed that the nsp3-4 molecular pore forms a large assembly with RTC components, including nsp3, nsp4, nsp7, nsp8, nsp9, nsp12, and nsp13, and confirmed that the replicase-transcriptase resides within the DMV interior, where viral RNA synthesis occurs. The single-stranded progeny RNA is likely exported to the cytoplasm through the molecular pore (18). Despite substantial advances in the structural characterization of DMVs, the molecular mechanisms underlying RTC component recruitment to DMVs and the roles of additional non-structural proteins in their biogenesis remain to be elucidated.

To dissect the viral protein-mediated mechanisms of DMV biogenesis, a surrogate system for DMV formation, independent of viral replication, provides a tractable platform for genetic manipulation of viral proteins and subsequent phenotypic analysis. In this study, we established an expression system for viral polyproteins spanning nsp3 to nsp10 to assess their ability to induce DMV formation. Previous studies have shown that co-expression of SARS-CoV-2 nsp3 and nsp4 alone is sufficient to induce DMV, with mean diameters of ~104 nm (15) or ~100 nm for nsp3/4 when expressed in *trans* (22). Consistent with these findings, we observed DMVs in cells expressing nsp3-4-GFP, with an average diameter of ~125 nm (Fig. 2B and D). Moreover, expression of nsp3-8-GFP and nsp3-10-GFP resulted in DMVs with larger average diameters than those induced by nsp3-4-GFP, and these vesicles exhibited greater morphological heterogeneity. These results suggest that additional viral proteins, namely nsp5-8 and nsp5-10, contribute to DMV biogenesis (Fig. 2B and C). Notably, DMVs formed in nsp3-10-GFP-expressing cells in our system were smaller than those observed during viral infection, where Cryo-ET measurements reported an average diameter of ~338 nm (17). This discrepancy may be attributed to the absence of other viral proteins or viral genomic RNA in the surrogate system.

In HCoV-229E-infected cells subjected to digitonin permeabilization, viral dsRNA and nsp7 were found to be inaccessible to antibodies in immunofluorescence assays, while nsp8 and nsp9 exhibited only weak accessibility, indicating that these RTC components are localized within DMV interior (18). In our surrogate system expressing nsp3-8-GFP and nsp3-10-GFP, the subcellular fractionation experiment showed that a portion of nsp5, nsp7, and nsp8 localized to DMV-associated membrane fractions (Fig. 3B and D) and displayed partial resistance to PK digestion (Fig. 3E through H), suggesting their incorporation into DMV interior or close association with DMV membrane architecture that shielded them from digestion.

Due to the limited availability of reliable antibodies, we could not assess nsp6 localization in relation to DMVs in our system. In a system co-expressing nsp3, nsp4, and nsp6, nsp6 formed structures resembling the “connectors” observed in infected cells (10), which mediate the zippering of the ER membrane (19). We identified a highly conserved MAE at the C-terminus of nsp6, which is crucial for viral replication (21). Introduction of a non-functional MAE mutant (MH) into the nsp3-10-GFP expression system selectively impaired nsp5 Mpro-mediated cleavage at the junctions downstream of nsp6, while leaving nsp3 PLpro-mediated cleavage and nsp5 Mpro-mediated cleavage upstream of nsp6 unaffected (Fig. 4B). This regulatory role of nsp6 MAE in polyprotein cleavage may be analogous to the function proposed for the N-terminal amphipathic helix of hepatitis C virus NS5A, which positions substrates on the membrane to facilitate protein–protein interactions and promote polyprotein cleavage at the NS5A/5B junction, but not at the NS4B/5A junction (41). DMV formation was markedly reduced in MH-expressing cells, which instead displayed extensive cross-linked ER regions (Fig. 4D). MH did not alter the PK digestion sensitivity of nsp5 or nsp8 (Fig. 4C), suggesting that the disordered ER structure itself may shield these proteins from digestion. Given nsp6’s role in ER membrane zippering to form connectors (19) and the importance of the MAE in polyprotein processing, the abnormal ER phenotype in MH cells may arise from defective ER connector formation, aberrant polyprotein processing that induces inappropriate viral protein–protein interactions, or both.

A recent study showed co-expression of nsp3, nsp4, and nsp12 enables interaction between the N-terminal domain of nsp3 and nsp12, facilitating nsp12 recruitment to DMVs (22). However, during authentic infection, DMV assembly and RTC components recruitment likely require polyprotein cleavage and subsequent cis-interactions among viral proteins (42, 43). Nsp3/nsp4 cleavage is essential for DMV formation in the nsp3-4 expression system, and cleavage site mutations result in multilayered paired-membrane structures instead of DMVs (15, 16). In contrast to these reports, we observed DMV-like structures with enlarged diameters in cells expressing catalytically inactive nsp3-4-GFP.C856A (Fig. 2B and D). This discrepancy might be due to the difference in mutation site: one targeting the substrate cleavage site, the other the enzymatic active site. Definitive structural characterization of these vesicles will require Cryo-ET.

We further examined the roles of PLpro- and Mpro-mediated cleavage events in DMV biogenesis using nsp3-8-GFP. Cells expressing nsp3-8-GFP.C856A failed to produce DMVs, instead forming extended membrane sheets (Fig. 5C). In contrast, nsp3-8-GFP.C145A-expressing cells formed DMVs with diameters comparable to wild type, though with increased morphological heterogeneity (Fig. 5C and D). Cleavage at the nsp3/4 junction and nsp5-mediated processing occurred independently (Fig. 5A; Fig. S4B). Under the nsp3-8-GFP.C856A condition, processed nsp5-8 may interact with nsp3-4 in a manner that disrupts membrane remodeling by uncleaved nsp3-4, leading to extended membrane sheets, rather than DMV-like structures in nsp3-4-GFP.C856A-expressing cells (Fig. 2B and 5C). In the nsp3-8-GFP.C145A context, DMV morphology resembled that of wild type, but whether uncleaved nsp4-8 is incorporated into the DMV interior remains to be elucidated.

In summary, we have developed a surrogate system for SARS-CoV-2 polyproteins that induces DMV formation, recapitulating key structural features observed during viral infection. Using this system, we have confirmed the critical roles of the nsp6 MAE in DMV biogenesis and investigated the interplay between viral polyprotein processing and DMV assembly. This platform offers a valuable tool for dissecting the molecular mechanisms by which nsp6 and other viral proteins contribute to DMV biogenesis.

## MATERIALS AND METHODS

### Plasmids

All SARS-CoV-2 sequences used in this study correspond to the nCoV-SH01 strain (accession number MT121215). The plasmid pLC-nCoV-A-FLAGnsp3-BsaI contains a cassette encoding a 3×FLAG tag (DYKDHD GDYKDH DIDYKD DDDK) and restriction sites for *BamHI* and *MluI* at the N-terminus of nsp3. Plasmid pLCCMV-nsp3 was constructed by stepwise cloning of the 3×FLAG-nsp3 sequence into the *EcoRI*/*NotI* site of a custom pLCCMV backbone, derived from the phCMV plasmid (Genlantis) by replacing its replication origin with a low-copy replication origin. The pLCCMV vector contains an ORF under the control of a CMV promoter with a CMV intron upstream of the ORF. Plasmids pLCCMV-GFP-nsp3 and pLCCMV-nsp3-GFP were created using an In-Fusion assembly (Hieff Clone Plus Multi One Step Cloning Kit; YEASEN, Cat. 10912), inserting the GFP coding sequence in-frame into *EcoRI*- or *NotI-*digested pLCCMV-nsp3, respectively. The nsp4 coding sequence was cloned into pLCCMV-Flag-nsp3-GFP via restriction-ligation to generate pLCCMV-FLAG-nsp3-4-GFP. Catalytically inactive mutants of the papain-like protease (nsp3PLpro, C856A) and the main protease (nsp5Mpro, C145A) were introduced by fusion PCR-mediated mutagenesis. Plasmids pLCCMV-nsp3-5-GFP, pLCCMV-nsp3-6-GFP, pLCCMV-nsp3-8-GFP, and pLCCMV-nsp3-10-GFP were constructed by restriction-ligation cloning. Codon-optimized sequences for nsp3-10 were synthesized by BGI (Wuxi). The plasmid phCMV-HA-optnsp5 was generated by amplifying the codon-optimized nsp5 region from Optnsp3-10, flanked by an N-terminal HA tag (YPYDVPDYA), and cloning the fragment into phCMV. Similarly, phCMV-FLAG-Optnsp6 was generated by amplifying the optimized nsp6 region with an N-terminal 3×FLAGtag. Plasmid phCMV-optnsp7-GFP was constructed by fusing the codon-optimized nsp7 sequence to EGFP and inserting the resulting fusion into the phCMV vector. The plasmids pLCCMV-nsp3-6-GFP.MH and pLCCMV-nsp3-10-GFP.MH were constructed by amplifying a fragment from pLC-nCoV-B-BsaI-nsp6C.MHmut (21) and cloning it into pLCCMV-nsp3-6-GFP and pLCCMV-nsp3-10-GFP using In-Fusion assembly. For control constructs, phCMV-GFP was generated by inserting the GFP coding sequence into the *XhoI*/*KpnI* sites of phCMV. Subsequently, phCMV-ER-GFP was constructed by fusing an endoplasmic reticulum-targeting transmembrane helix derived from p450 (N29) to the N-terminus of GFP and appending a C-terminal 3×Flag tag, followed by cloning into the *BglII*/*NotI* sites of phCMV. To create phCMV-ER-msfGFP, the GFP sequence in phCMV-ER-GFP was replaced with the msfGFP sequence (44). All plasmids were confirmed by Sanger sequencing, and the complete sequence information is available upon request.

### Cells

The human embryonic kidney cell line HEK-293T and the human hepatocellular carcinoma cell line Huh7 cells were obtained from the Cell Bank of the Chinese Academy of Sciences (https://www.cellbank.org.cn/). Cells were cultured in Dulbecco's modified medium containing 10% fetal bovine serum (FBS, Gibco and Vazyme) at 37°C in a humidified incubator with 5% CO_2_.

### Antibodies

The following antibodies were used for western blotting: Anti-GFP (Santa Cruz, sc-9996; 1:1,000), anti-FLAG (Sigma, F1804; 1:2,000), anti-β-actin (Affinity, T0022; 1:3,000), anti-Calnexin (BD, 610524; 1:2,000), anti-Hsp70 (ABclonal, A23457; 1:1,000), and anti-GAPDH (Affinity, AF7021; 1:10,000). SARS-CoV-2-specific antibodies, including anti-nsp5 (GeneTex, GTX636807; 1:2,000), anti-nsp6 antibody (ProSci, 9177; 1:2,000), anti-nsp7 (GeneTex, GTX636719; 1:2,000), and anti-nsp8 antibody (GeneTex, GTX632696; 1:2,000), were used for western blotting. Anti-Mouse IgG HRP (CST, 7076) and anti-Rabbit IgG HRP (CST, 7074) were used at a dilution of 1:5,000 in western blotting.

### Transfection

For plasmid transfection, 9.0 × 10^5^ HEK293T cells were seeded onto poly-L-lysine-coated 6-well plates. The following day, 2 µg of plasmid DNA was transfected using Lipofectamine 3000 (Invitrogen, L3000015) according to the manufacturer’s instructions. Alternatively, 7.0 × 10^6^ HEK293T cells seeded in a 10 cm dish and 2.25 × 10^5^ cells seeded in a 24-well plate were transfected with 10 and 0.5 µg of plasmid DNA, respectively, following the same procedure.

### Fluorescence

Cells were fixed in 4% paraformaldehyde in PBS for 10 min at room temperature, washed three times with PBS, and subsequently imaged using a fluorescence microscope (EVOSM500, Thermo Fisher Scientific).

### Flow cytometry

Cells were trypsinized at room temperature, resuspended in PBS containing 2% FBS and 4% paraformaldehyde, and fixed for 20 min. The cell suspension was passed through a 70 µm cell strainer, and GFP-positive cells were quantified using the FITC channel of a flow cytometer (Attune NxT, Thermo Fisher Scientific).

### Cellular fractionation

HEK293T cells were plated in 6-well plates and transfected as described. Cells were permeabilized by incubation with 0.4 mL digitonin solution (50 µg/mL; Merck, 300410) in PK buffer (20 mM HEPES, 110 mM potassium acetate, 2 mM magnesium acetate, 1 mM EDTA) at room temperature for 5 min. The cytosolic fraction (c1) was obtained from the supernatant, and proteins were precipitated by adding four volumes of methanol, followed by centrifugation at 12,000 × *g* for 10 min. The resulting pellet was resuspended in 30 µL 2× SDS loading buffer (100 mM Tris-Cl [pH 6.8], 4% SDS, 0.2% bromophenol blue, 20% glycerol, and 10% 2-mercaptoethanol). The membrane fraction (m1), comprising the remaining cellular material, was resuspended in 30 µL 2× SDS loading buffer for further analysis.

Alternatively, cellular fractionation was performed following a modified version of a previously described protocol (33). Cells cultured in 10 cm dishes were rinsed once with 3 mL of ice-cold PBS, scraped into 1 mL of PBS, and pelleted by centrifugation at 900 × *g* for 5 min at 4°C. Pellets were resuspended in 330 µL hypotonic lysis buffer (20 mM Tris, pH 8.0, 10 mM sodium acetate, 1.5 mM MgCl₂) and incubated on ice for 30 min. Cells were homogenized by 25 passages through a 1 mL syringe and centrifuged at 900 × *g* for 5 min at 4°C to remove nuclei and debris. The post-nuclear supernatant was centrifuged again at 900 × *g* for 5 min at 4°C. A 100 µL aliquot of the supernatant was further centrifuged at 15,000 × *g* for 20 min at 4°C to yield the cytoplasmic fraction (c2) in the supernatant and the crude membrane fraction (m2) in the pellet. Proteins from the cytoplasmic fraction were precipitated with four volumes of ice-cold methanol and pelleted by centrifugation at 12,000 × *g* for 10 min at 4°C, then resuspended in 40 µL 2× SDS loading buffer and sonicated. The crude membrane fraction was washed once with hypotonic buffer, pelleted at 15,000 × *g* for 20 min at 4°C, resuspended in 40 µL 2× SDS loading buffer, and sonicated.

### Proteinase K digestion

Cultured cells in 6-well plates were rinsed once in PBS, permeabilization by treatment with 0.4 mL digitonin solution (50 µg/mL) in PK buffer at room temperature for 5 min. Cells were then harvested by scraping into 400 µL PK buffer and incubated with or without proteinase K (10 µg/mL, Ambion) at 37°C for 5 min. Digestion was terminated, and proteins were precipitated by adding an equal volume of 40% trichloroacetic acid (TCA), followed by incubation on ice for 30 min. Samples were centrifuged at 12,000 × *g* for 10 min, and the resulting protein pellets were washed with 500 µL acetone and reconstituted in 30 µL lysis buffer (7 M urea, 2 M thiourea). Protein solutions were mixed with an equal volume of 2× SDS loading buffer, boiled for 5 min, and analyzed by western blotting.

### Western blotting

Following PBS washes, cells were lysed in 2× SDS loading buffer. Lysates were aspirated 20 times with a 1 mL syringe to achieve a fluid consistency and were loaded without boiling to enable detection of nsp3- and nsp4-containing polypeptides. In contrast, samples from proteinase K digestion and cellular fractionation experiments were resuspended in 2× SDS loading buffer and boiled for 5 min prior to electrophoresis. Proteins were separated by SDS-PAGE and transferred onto a nitrocellulose membrane. Membranes were blocked for 1 h in PBS containing 5% milk and 0.05% Tween-20, then incubated with the primary antibodies diluted in the blocking buffer. After three washes with PBST (PBS containing 0.05% Tween-20), membranes were incubated with appropriate secondary antibodies, followed by another three washes with PBST. Protein bands were visualized using Western Lightning Plus-ECL substrate (PerkinElmer, NEL10500) or the Odyssey CLx Imaging System. If necessary, band intensities were quantified by densitometry using ImageJ software.

### Luciferase activity

To assess SARS-CoV-2 replicon replication, 10 µL of supernatant from replicon RNA-transfected cells was mixed with an equal volume of 2× passive lysis buffer (Promega, E2820) and analyzed for luciferase activity using the Renilla luciferase substrate (Promega, E2820) according to the manufacturer’s instructions.

### Electron microscopy

Transfected 293T cells were fixed sequentially in 2.5% glutaraldehyde for 20 min at room temperature, followed by an additional fixation for 1 h at 4°C. Cells were washed three times (3 min each) with 0.1 M phosphate buffer (PB; 0.2 M NaH_2_PO_4_, 0.2 M Na_2_HPO_4_, pH [7.4]), then incubated at 4°C in the dark for 30 min with a 1:1 mixture of 2% osmium tetroxide (OsO_4_) and 3% potassium hexacyanoferrate (II) trihydrate (Sigma, P3289). After three washes with double-distilled water (ddH_2_O), cells were treated with 1% thiocarbohydrazide (TCH; Sigma, 223220) for 30 min at room temperature in the dark, washed again with ddH_2_O, and post-fixed with 1% osmium tetroxide for 30 min at room temperature in the dark. Following ddH_2_O washes, samples were incubated overnight at room temperature in 2% uranyl acetate, then placed in a 37°C oven for 1 h. Cells were dehydrated through a graded ethanol series (30%, 50%, 70%, 85%, 95%; 5 min each), followed by two washes in 100% ethanol (10 min each) at room temperature. Samples were infiltrated stepwise with anhydrous ethanol: epon812 resin (Ted Pella, Inc. Eponate, 18010) mixture at ratios of 3:1, 1:1, and 1:3 (45 min each), then with pure resin overnight. The following day, fresh resin was added and incubated for 2 h at room temperature. Pre-polymerized resin columns were inverted onto the cell culture dish, and polymerization was performed at 65°C for 48 h. Polymerized resin micropillars were trimmed on a Leica EM UC7 ultramicrotome and sectioned into 70 nm ultrathin slices, which were mounted on copper grids (Zhongjingkeyi Technology, BZ11221Sa). Sections were imaged using a Talos L120C TEM under high-vacuum conditions, following the manufacturer’s operating guidelines. Imaging parameters included an accelerating voltage of 120 kV, filament temperature of 35°C, emission current of 5.00 μA, and a high-contrast mode to enhance structural resolution. Two or three independent experiments were performed for each sample, with 2–3 sections prepared per experiment. All sections were examined manually, and images containing DMV-positive cells were acquired. An equivalent number of images was acquired for the nsp3-4-GFP group to ensure consistency. Vesicle diameters were measured using ImageJ when applicable.

### Statistical analysis

All results were derived from 2 to 4 independent experiments. Comparison between two groups was performed using an unpaired, two-tailed Student’s *t*-test, or the Mann-Whitney test for small sample sizes. Multiple group comparisons were conducted using one-way analysis of variance (ANOVA), followed by Dunnett’s multiple comparisons test for pairwise analysis. Statistical analyses were conducted using the GraphPad Prism 8 (GraphPad Software). A *P* < 0.05 was considered statistically significant. Significance levels are indicated as follows: ns, *P* ≥ 0.05; *, *P* < 0.05; **, *P* < 0.01; ***, *P* < 0.001. The specific tests applied are detailed in the figure legends.

## Data Availability

All data generated during this study are included in the article. Additional data sets are available from the corresponding author upon reasonable request.

## SUPPLEMENTAL MATERIAL

Supplemental figures jvi.01385-25-s0001.pdf Figures S1 to S6.

ASM does not own the copyrights to Supplemental Material that may be linked to, or accessed through, an article. The authors have granted ASM a non-exclusive, world-wide license to publish the Supplemental Material files. Please contact the corresponding author directly for reuse.
