# Supplementary material for: SARS-CoV-2 polyprotein expression and the induction of double-membrane vesicles
Source: J Virol. 2025 Nov 6;99(11):e01385-25. doi: 10.1128/jvi.01385-25 (PMC12645958; doi:10.1128/jvi.01385-25)
Supplement: Supplemental figures — Figures S1 to S6. [file jvi.01385-25-s0001.pdf]

Supplementary Figure 1

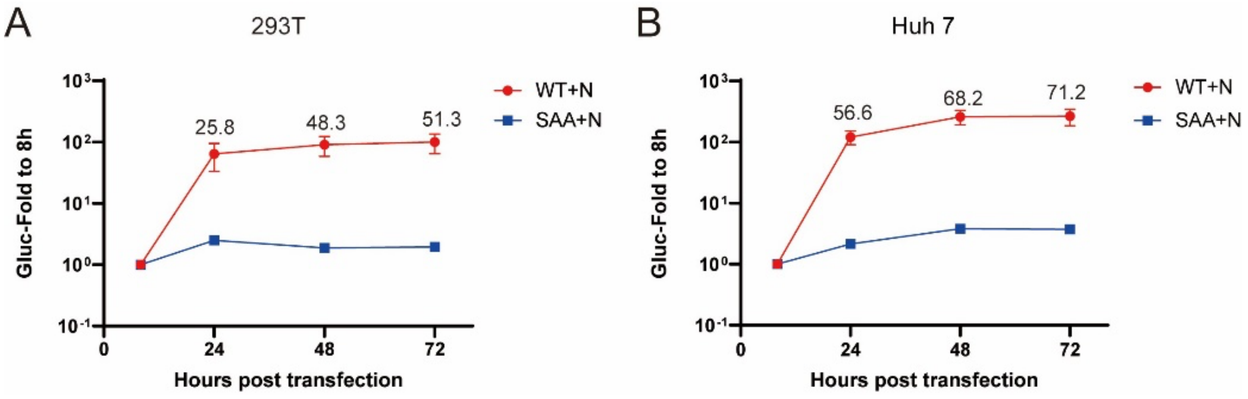

Figure S1. Replication kinetics of the SARS-CoV-2 replicon in HEK293T and Huh7 cells. HEK293T cells (A) and Huh7 (B) were co-transfected with in vitro-transcribed SARS-CoV-2 replicon RNAs and an mRNA encoding the nucleocapsid (N) protein. The culture medium was replaced 8 h post-transfection. Luciferase activity in the supernatants was measured at the indicated time points. A replication-deficient nsp12 polymerase active-site mutant (SAA) served as a negative control. Data were normalized to luciferase activity at 8 h post-transfection and are presented as mean ± standard deviation (SD) from triplicate wells. Fold differences in luciferase activity between WT group and SAA groups at each time point are indicated. Similar results were obtained in an independent experiment.

Supplementary Figure 2

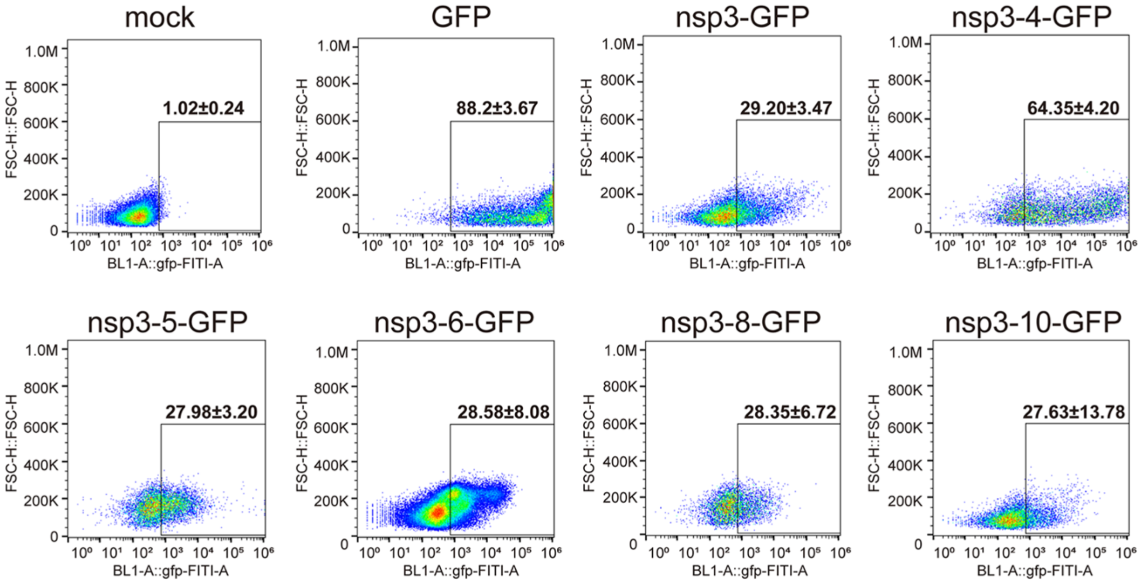

Figure S2. Expression analysis of SARS-CoV-2 polyprotein constructs. HEK293T cells were transfected with the indicated plasmids and analyzed 48 h post-transfection. The percentage of GFP-positive cells was quantified by flow cytometry. Data are represented as mean ± standard error of the mean (SEM), with n = 4. Representative results from two independent experiments are shown.

Supplementary Figure 3

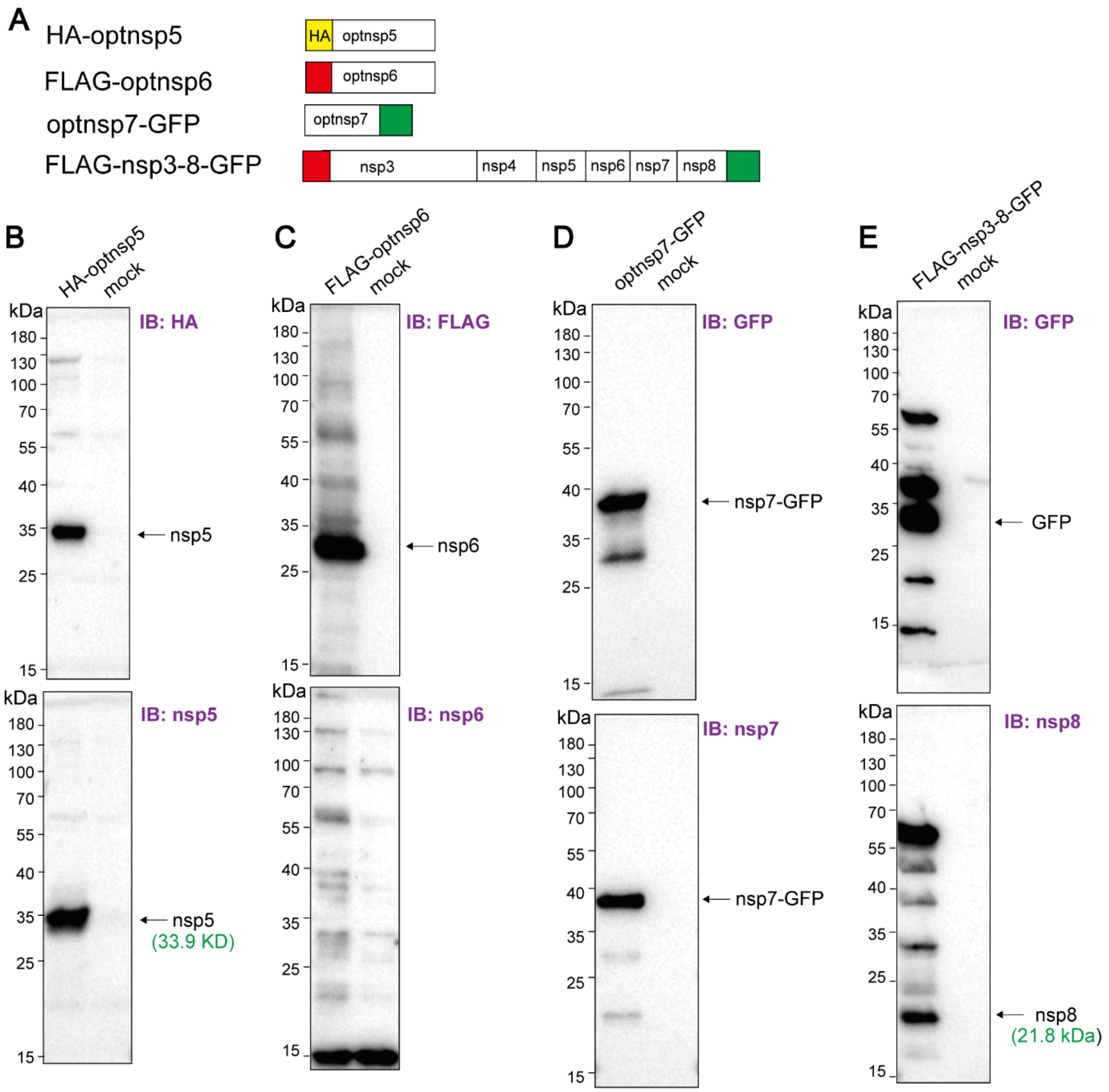

Figure S3. Detection of antibodies against SARS-CoV-2 nonstructural proteins. (A) Schematic diagrams of HA-optnsp5, FLAG-optnsp6, optnsp7-GFP, and nsp3-8-GFP constructs. (B–E) HEK293T cells were transfected with HA-optnsp5, FLAG-optnsp6, optnsp7-GFP, or nsp3-8-GFP plasmids to evaluate antibody specificity against nsp5, nsp6, nsp7, and nsp8. Protein expression was analyzed 48 h post-transfection by Western blotting using target-specific or anti-tag antibodies (immunoblotting, IB). Specific protein bands are indicated by arrows. Molecular weight markers (in kDa) are labeled on the left.

Supplementary Figure 4

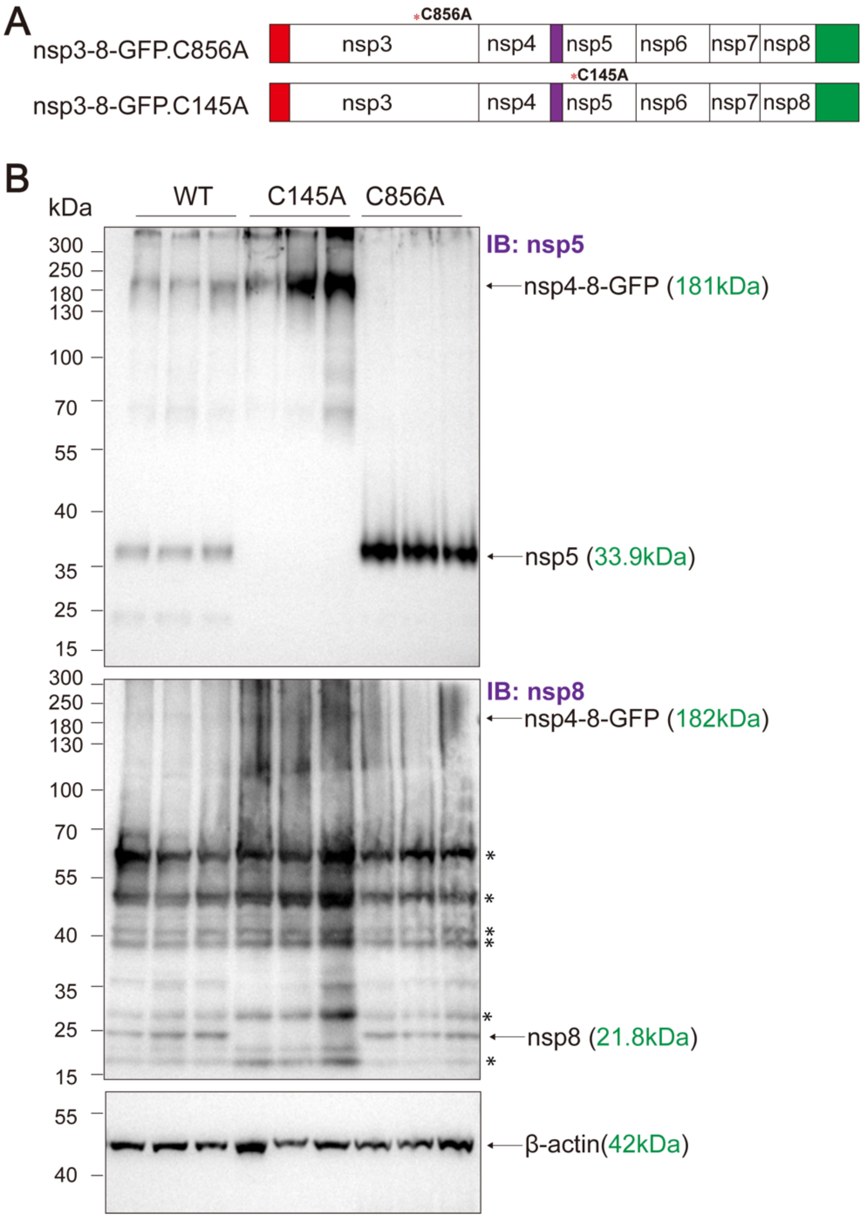

Figure S4. Cleavage of nsp3 PLpro and nsp5 Mpro inactivation mutants in nsp3-8-GFP constructs. (A) Schematic diagrams of constructs: C856A, nsp3 PLpro inactivation mutant; C145A, nsp5 Mpro inactivation mutant. (B) HEK293T cells were transfected with plasmids encoding nsp3-8-GFP (WT), nsp3-GFP.C145A (C145A), or nsp3-8-GFP.C856A (C856A). Protein expression was analyzed 48 h post-transfection by Western blotting using the indicated antibodies (immunoblotting, IB). Specific protein bands are indicated by arrows. Unspecified bands are marked with asterisk. Molecular weight markers (in kDa) are shown on the left.

Supplementary Figure 5

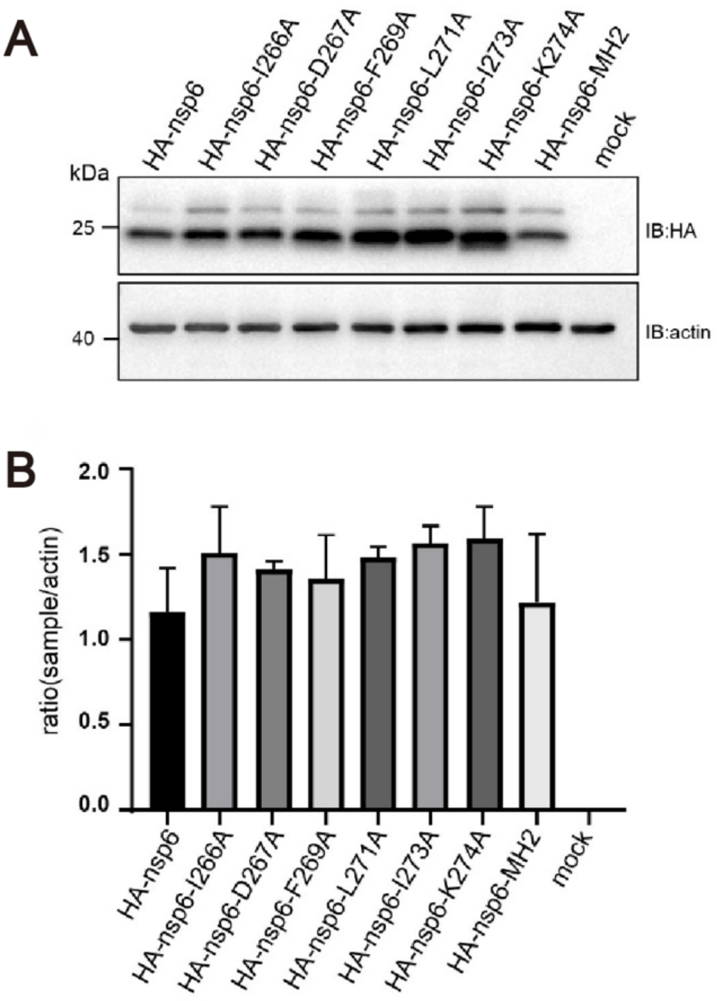

Figure S5. Effects of MH mutations on nsp6 expression. (A) HEK293T cells were transfected with HA-nsp6 or HA-nsp6 mutants and analyzed 48 h post-transfection by Western blotting using the indicated antibodies (IB). (B) Band intensities of HA-nsp6 variants and Actin were quantified using ImageJ software (mean ± SD, n = 3). No significant differences from WT were observed (Student's two-tailed *t*-test).

Supplementary Figure 6

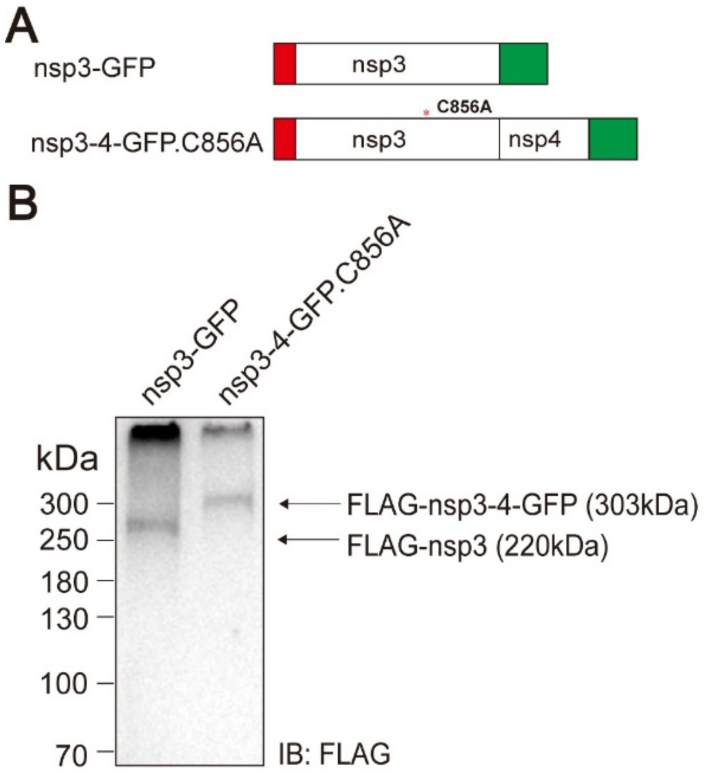

Figure S6. Cleavage of the nsp3 PLpro inactivation mutant. (A) Schematic diagram of the C856A nsp3 PLpro inactivation mutant. (B) HEK293T cells were transiently transfected with plasmids expressing nsp3-GFP or nsp3-4-GFP.C856A. Protein expression was analyzed 48 h post-transfection by Western blotting using the indicated antibodies (immunoblotting, IB). A representative blot from multiple independent experiments is shown.
